# Cycling the Breath in Noninvasive Home Ventilation

**DOI:** 10.3390/jcm13226673

**Published:** 2024-11-06

**Authors:** Jean-Michel Arnal, Sonia Khirani

**Affiliations:** 1 Intensive Care Unit and Home Ventilation Unit, Hôpital Sainte Musse, 83100 Toulon, France; jean-michel@arnal.org; 2 ASV Santé, 92230 Gennevilliers, France; 3 Pediatric Noninvasive Ventilation and Sleep Unit, AP-HP Hôpital Necker-Enfants Malades, 75015 Paris, France; 4 EA 7330 VIFASOM, Université de Paris Cité, 75006 Paris, France

**Keywords:** spontaneous/timed mode, cycling, expiratory trigger sensitivity, inspiratory time, leaks

## Abstract

Spontaneous/timed (ST) mode is widely used for long-term noninvasive ventilation (NIV) in adults and children. It combines controlled, assisted, and spontaneous breaths. Cycling refers to the switch from inspiration to exhalation. In ST mode, different cycling mechanisms coexist. In spontaneous breathing, cycling is set by the expiratory trigger sensitivity (TgE) based on the inspiratory flow signal, which results in variable inspiratory times (Ti) and appears to be more physiological. In the case of controlled breathing or unintentional leaks, the cycling is time-dependent according to the set backup Ti or Ti max, respectively. Cycling is an important parameter to set adequately to avoid patient–ventilator asynchronies. This article gathers all the information about cycling in long-term NIV, presenting the cycling settings for different devices, addressing cycling issues, and detailing how to set the cycling criteria. Advanced monitoring with statistics and waveforms is discussed to detect early and delayed cycling.

## 1. Introduction

In mechanical ventilation, cycling refers to the switch from inspiration to exhalation. For a ventilator, the signal to end mechanical insufflation is based either on time, flow, pressure, or, rarely, inspiratory volume. In controlled and assisted-controlled modes, cycling is based on the inspiratory time (Ti) set by the user [1,2]. Therefore, the Ti is fixed and may not always match the patient’s neural time. In spontaneous modes, such as pressure support, cycling is based on flow, which results in a variable Ti [1,2]. A variable Ti offers a major advantage because it reproduces normal non-assisted breaths and enhances phase synchronization. Patient–ventilator synchronization is very important in noninvasive ventilation (NIV) to insure tolerance, comfort, efficacy, and sleep quality [3,4].

Long-term home NIV is widely used in adults and children, with the objective to correct nocturnal alveolar hypoventilation in patients presenting different etiologies [5]. The most widely used ventilatory mode is spontaneous/timed (ST) mode, which combines controlled, assisted, and spontaneous breaths [6,7]. Therefore, in the same mode, different cycling mechanisms coexist. Cycling is also affected by unintentional leaks and the settings of the backup Ti or the minimal (Ti min) and maximal (Ti max) Ti. Therefore, the ventilator settings have a major influence on the cycling and should be individualized to optimize patient–ventilator synchrony.

This report gathers the available data on the cycling settings in ST mode for different devices and manufacturers and reviews the cycling strategies proposed in the case of unintentional leaks. This report also addresses troubleshooting related to inadequate cycling setting.

## 2. Cycling in ST Mode

**Figure 1:**
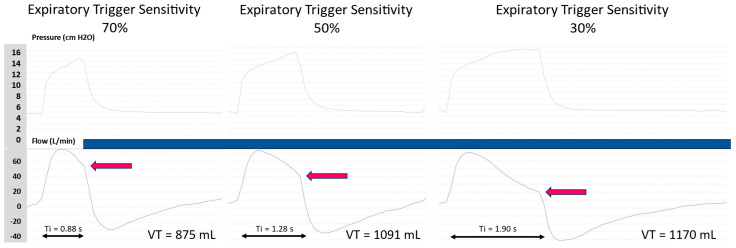
The effect of the expiratory trigger sensitivity (TgE) on inspiratory time and tidal volume. Red arrows indicate the cycling flow. When the TgE is set as a percentage of the peak inspiratory flow, a high percentage of TgE leads to a short inspiratory time (left panel). When the TgE percentage is decreased, the actual inspiratory time (Ti) and the tidal volume (VT) increase.

In ST mode, a spontaneous breath cycle is set by the user according to a flow cycling criterion called the expiratory trigger sensitivity (TgE). In most devices, the user sets the TgE as a fixed percentage of the peak inspiratory flow. During each inspiration, the inspiratory flow decreases and the ventilator cycles when the inspiratory flow reaches the threshold set by the user according to the following formula: Cycling flow (L/min) = Peak inspiratory flow (L/min) × TgE (%). Therefore, a high percentage of TgE results in a short Ti, while a low percentage of TgE results in a longer Ti (Figure 1). In some devices, the TgE is set as a percentage of the diminution of the peak inspiratory flow; therefore, a low percentage of TgE will result in a short Ti. The same cycling criteria apply for automatic modes with a guaranteed tidal volume and/or an automatic expiratory positive airway pressure (EPAP), such as AVAPS, iVAPS, AVAPS-AE, autoST, and EFL.

The actual Ti depends on the peak inspiratory flow and the shape of the decreasing inspiratory flow. The peak inspiratory flow depends on the pressure support, pressure rise time, patient effort, and respiratory mechanics. The shape of the decreasing inspiratory flow depends mainly on the respiratory mechanics but also on the patient’s effort, if any. Consequently, because the patient’s effort and respiratory mechanics change from breath to breath or during the nighttime, a fixed setting of the TgE results in a variable Ti. The TgE is presented according to the manufacturers as a percentage of the peak inspiratory flow, or using a numerical scale (1–9) or a word rating scale (high, medium, and low levels), which corresponds to certain predefined percentages. The range of the TgE depends on the device, with some devices offering large ranges (from 90% to 10% of the peak inspiratory flow) and others offering narrower ranges (from 50% to 8%) (Table 1). Some manufacturers also offer an “auto” setting. The setting is a fixed setting by default for Löwenstein (Bad Ems, Germany) and Breas (Mölnlycke, Sweden) ventilators, while it is a true automatic setting that varies according to the patient’s breathing for Philips (Best, The Netherlands) and Air Liquide (Anthony, France) ventilators. Philips ventilators allow the operator to set an automated cycling, named auto-trak, that combines several mechanisms for cycling: a decrease in inspiratory flow of 15 L/min within 300 ms, a reverse in the shape of inspiratory flow, a Ti max, and an algorithm based on the shape of the inspiratory flow rise. As soon as one of these criteria is achieved, the breath cycles, which is a way to prevent delayed cycling under different conditions of respiratory mechanics and unintentional leaks. Air Liquide ventilators offer automatic cycling based on the peak inspiratory flow and an arbitrary Ti max. A correct understanding of the different definitions of the TgE may prevent inappropriate settings.

When a patient-triggered breath is cycled by the Ti min or Ti max, it is then an assisted breath with a fixed Ti. When the breath is triggered by the backup respiratory rate (RR), the Ti is fixed in most of the manufacturers’ ventilators and is set by the user. This is called the backup Ti or Ti timed. However, some manufacturers still use the TgE to cycle controlled breathing within the limits of the Ti min and Ti max. Table 1 shows the mechanisms to cycle controlled breathing according to the ventilators.

## 3. How to Set Cycling

**Figure 2:**
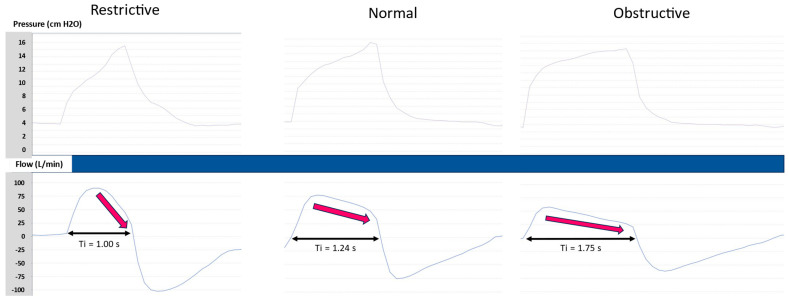
The effect of the respiratory mechanics on the inspiratory time (Ti). Because the shape of the inspiratory flow depends on the respiratory mechanics (red arrows), a given setting of the expiratory trigger sensitivity (40% of the peak inspiratory flow) is associated with a short Ti for restrictive mechanics and a long Ti for obstructive mechanics.

The TgE is an important setting affecting patient–ventilator synchrony, comfort, and, eventually, tolerance. The goal is to provide an insufflation time that matches the patient’s neural Ti and prevents both early and delayed cycling. Both of these asynchronies can be identified by clinical inspection, reported by the patient, and detected on the pressure and flow waveforms [8]. In practice, the TgE is adjusted during the NIV titration session. To start, the Ti min should be set very short, Ti max very long, and backup RR very low to be sure that each breath is triggered by the patient and cycled by the TgE. After setting an adequate EPAP, inspiratory positive airway pressure (IPAP), and pressure rise time, different TgEs are tested for the patient in order to select the most comfortable setting. If the patient is not able to choose the optimal setting, the clinician can use clinical inspection and waveform interpretation to decide. The setting of the TgE can be guided by the underlying conditions. Indeed, the shape of the inspiratory flow curve is influenced by the patient’s respiratory mechanics [9,10]. In restrictive patients, the inspiratory flow decay is rapid due to low lung and/or chest wall compliance. The TgE should therefore be set below 30% to allow for a minimum Ti. In obstructive patients, the flow decay is progressive due to the high inspiratory resistance. The TgE should therefore be set above 50% to prevent a long Ti and allow for a sufficient expiratory time (Figure 2).

The resulting Ti also depends on the RR. A spontaneous RR is physiologically dependent on CO_2_ production, which imposes the minute ventilation, the anatomical dead space, and the respiratory mechanics [11,12]. Consequently, obstructive patients select a low RR with a large tidal volume (VT) and a long Ti, while restrictive patients select a high RR with a low VT and a short Ti.

After selecting the optimal TgE, clinicians should monitor the actual RR and Ti and use those as references to set the Ti min, Ti max, backup Ti or Ti timed, and backup RR. The backup Ti or Ti timed is usually set close to the actual daytime Ti, assuming that the patient requires the same Ti when the breath is triggered by the patient or by the backup RR.

## 4. How to Set Ti Min and Ti Max

**Figure 3:**
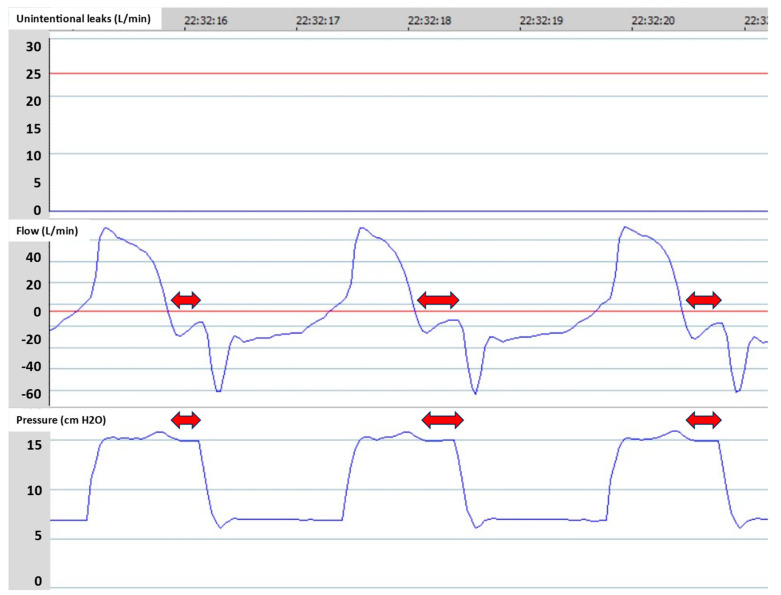
Delayed cycling due to a too long minimal inspiratory time (Ti min). In this example, Ti min is set at 1 s. Because the inspiratory flow reaches the baseline before 1 s, there is a kind of plateau with no flow at the end of the insufflation before exhalation (red arrows). This prolonged insufflation time beyond the patient’s neural time is likely to induce discomfort. Red arrows indicate the excess of time due to Ti min that induce the asynchrony.

The flow cycling mechanism using the TgE necessarily takes place in a time window between the Ti min and Ti max. Currently, most ventilators allow the operator to set a Ti min and Ti max. The Ti min is usually set very short, such as 0.2 s, as the Ti mainly depends on the TgE. Setting a longer Ti min exposes the patient to more discomfort in the case of autotriggering. However, severely restrictive patients may experience too short breaths with the TgE set at the lowest percentage possible, which is usually 5–10% of the peak inspiratory flow. In those cases, the Ti min can be set a little bit longer than their actual Ti to allow the inspiratory flow to reach the baseline. Prolonging the Ti min beyond this point is useless, as there is no more inspiratory flow and a risk of discomfort, as the patient may attempt to actively exhale without success (Figure 3).

**Figure 4:**
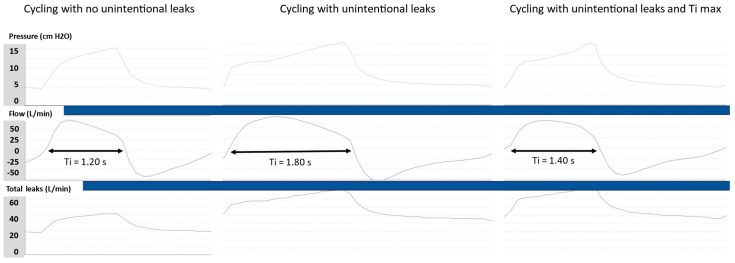
The effect of unintentional leaks on cycling. Unintentional leaks affect the shape of the inspiratory flow. Therefore, the time to reach the expiratory trigger threshold is increased, which leads to delayed cycling (**middle** panel). Setting a maximal inspiratory time (Ti max) slightly above the normal Ti (**left** panel) prevents delayed cycling (**right** panel).

Conversely, the Ti max is a very important setting, because the TgE fails to cycle the breath when there are significant unintentional leaks [13]. In the case of unintentional leaks, which are common in NIV, the inspiratory flow is distorted because part of the flow goes to the leak. This is recognized by a kind of plateau of the inspiratory flow at the end of the inspiration, which matches the unintentional leak rate. The consequence is that the flow does not reach the TgE threshold, and the Ti is prolonged. This results in delayed cycling that causes discomfort (Figure 4). The Ti max is used to prevent delayed cycling in the case of unintentional leaks. The Ti max is usually set at 0.2 s above the actual Ti without leaks in order to preserve the normal Ti variability and to prevent delayed cycling (Figure 4). However, in the case of a too short Ti max, all insufflations will have the same duration, and this will potentially generate early cycling asynchronies, as the patient will not receive enough VT and will attempt to prolong insufflation.

Minimal and maximal Ti default values are usually 0.2–0.3 and 2–3 s, respectively, depending on the device. The maximal Ti may be too long for children and should therefore always be adapted manually and not set at the default value.

In ventilators that do not allow the operator to set a Ti min and Ti max, cycling asynchronies may be more frequent in the case of restrictive diseases (short breaths) or unintentional leaks (delayed cycling) [14]. In these ventilators, the Ti cannot be longer than 3 s for safety reasons [14], and this cannot be modified, which may not be adequate, particularly for young children.

## 5. Troubleshooting with Cycling

**Figure 5:**
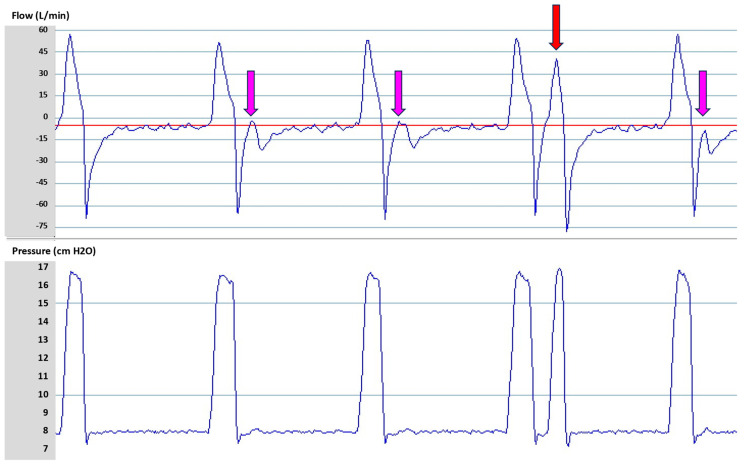
Early cycling (pink arrow) and double-triggering (red arrow). In this patient with severe restrictive respiratory mechanics, the expiratory trigger sensitivity is set at 25%. Early cycling is suspected on the second, third, and fifth breaths based on the distortion of the expiratory flow occurring very early after the start of exhalation (pink arrow). The fourth breath shows double-triggering (red arrow). Because of the severe early cycling, the patient triggers a second breath during the same inspiratory effort. Red line represents the flow baseline.

Early cycling may be due to a too short TgE setting (a too high percentage of peak inspiratory flow), a too short Ti min, or a too short Ti max (Table 2). Early cycling may cause discomfort, decrease the VT, and increase the RR, and, in the worst-case scenario, it may lead to double-triggering (Figure 5). Early cycling occurs mainly in restrictive patients. It is common that early cycling appears during the night when compliance transiently decreases as a result of the position and/or sleep stage.

**Figure 6:**
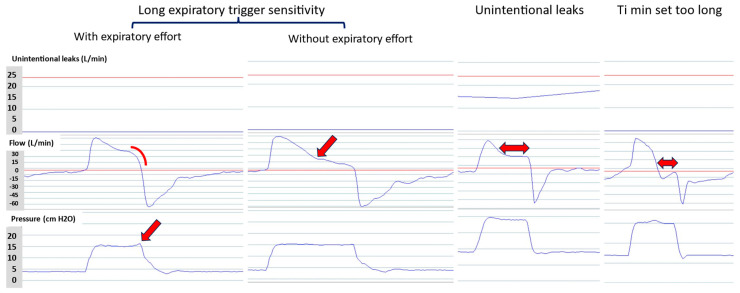
Causes of delayed cycling. Delayed cycling can be due to a too low expiratory trigger sensitivity (left panel), unintentional leaks (middle panel), or a too long minimal inspiratory time (Ti min) (right panel). Red arrows and line indicate the asynchrony.

Conversely, delayed cycling may be due to a long TgE setting (a too low percentage of peak inspiratory flow), unintentional leaks, or a too long Ti min (Table 2) [15]. The cause can be determined based on the waveform (Figure 6). Delayed cycling is usually poorly tolerated, increases the VT, and reduces the expiratory time, with a risk of worsened intrinsic positive end-expiratory pressure in obstructive patients.

When patients complain or waveforms demonstrate early or delayed cycling, the general strategy is to adjust the TgE setting first and the Ti max after. As an example, if a patient reports delayed cycling or if this is observed, the TgE should be shortened. Consequently, the actual Ti will decrease. Therefore, the Ti max should also be shortened to keep the 0.2 s delay between the actual Ti and the Ti max. The only exception is when the TgE reaches its limits of settings. Then, the Ti min or Ti max needs to be adjusted. For example, if the TgE is set at the lowest value and the patient still complains of early cycling, the Ti min can be used to increase the actual Ti a little bit. Conversely, if the TgE is set at the highest value and the patient complains of delayed cycling, then the Ti max can be used to shorten the actual Ti.

## 6. Monitoring the Cycling

**Figure 7:**
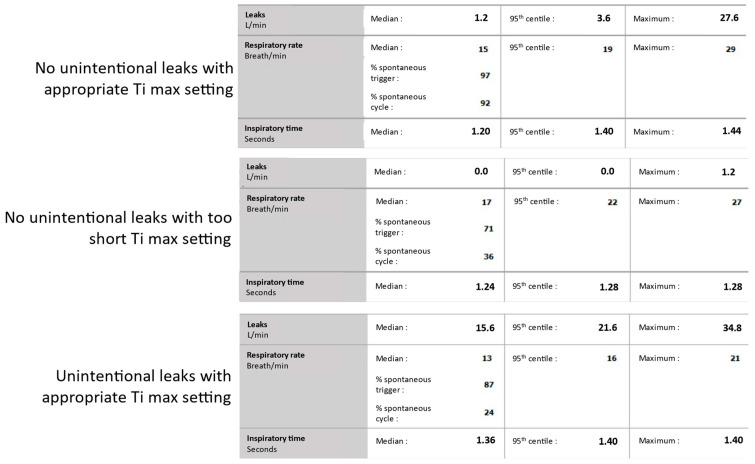
Monitoring cycling in spontaneous/timed mode. The available statistics on the built-in software provide information on unintentional leaks, the % of spontaneous cycle, and the actual inspiratory time (Ti). In the absence of unintentional leaks, most of the triggered breaths should be cycled by the expiratory trigger. The % of spontaneous cycling should therefore be above 80%, and there should be 0.2–0.3 s between the median Ti and the Ti max (upper panel). If the % of spontaneous cycling is below 50%, it suggests that the Ti max is set too short. Therefore, the median Ti is close to the Ti max (middle panel). In the case of significant unintentional leaks, the % of spontaneous cycling is low, because most of the breaths are cycled by the Ti max. Therefore, the median Ti is close to the Ti max (lower panel).

The correct setting of the Ti max can be assessed in some ventilators by analyzing the statistics reporting the unintentional leaks and the percentage of breaths cycled by the patient, i.e., using the TgE [1,16]. In the absence of unintentional leaks, most of the triggered breaths should be cycled by the TgE, and the median Ti should be 0.2–0.3 s below the Ti max (when the Ti max is set at 0.2–0.3 s over the patient’s Ti). In the case of significant unintentional leaks during the night, it is expected that most of the triggered breaths are cycled by the Ti max. Therefore, the median Ti is close to the Ti max (Figure 7).

## 7. Conclusions

Cycling in ST mode is an important setting affecting patient–ventilator synchronization and comfort. The goal is to favor the TgE in order to obtain a variable Ti and avoid the use of a fixed Ti. In the case of large unintentional leaks, the TgE fails, and the appropriate setting of the Ti max prevents delayed cycling. In restrictive patients, an adequate setting of the Ti min may be required to avoid short cycles. For ventilators that require setting a backup Ti or Ti timed, this should be set close to the normal Ti of the patient. Advanced monitoring with statistics and waveforms helps to readjust the settings and detect patient–ventilator asynchronies, such as early or delayed cycling.

## Figures and Tables

**Table 1:** The names and ranges of the expiratory trigger sensitivity (TgE) and the mechanisms of cycling controlled breathing in spontaneous/timed mode for the different ventilators.

Manufacturer	Ventilator	Name of TgE	Ranges of TgE (From Shortest to Longest Inspiration)	Ti Settings	Cycling of Controlled Breathing
ResMed (San Diego, CA, USA)	Lumis Stellar	Cycle	Very High (50%) to Very Low (8%)	Ti Min and Ti Max	According to Cycle within the limit between Ti Min and Ti Max
	Astral	Cycle	90% to 5%	Ti Min and Ti Max	According to Cycle within the limit between Ti Min and Ti Max
Löwenstein (Bad Ems, Germany)	prisma30ST	Trigger EX	70% to 30% or Auto = 50%	Ti Min and Ti Max Ti Timed	Ti Timed
	prisma VENT 40/50	Trigger Exhalation	95% to 5% Auto = 40%	Ti Min and Ti Max Ti Timed	Ti Timed or Trigger Exhalation if Ti Timed set on Auto
	Luisa	Exp. Sensitivity	95% to 5%	Ti Min and Ti Max Ti Timed	Ti Timed or Exp. Sensitivity if Ti Timed set on Auto
Breas (Mölnlycke, Sweden)	Vivo 1/2/3	Exp. Trigger	90% to 10% or Auto = 70% ∫	Min Insp. Time and Max Insp. Time Backup Insp. Time	Backup Insp. Time
	Vivo 45/55/65	Exp. Trigger	90% to 10% ∫	Min Insp. Time and Max Insp. Time (optional) Backup Insp. Time	Backup Insp. Time
Philips (Best, The Netherlands)	BiPAP A40	Flow Cycle Sensitivity or Auto-trak	90% to 10% or Auto-trak *	Inspiratory Time	Inspiratory Time
	Trilogy Evo	Flow Cycle Sens. or Auto-trak	90% to 10% or Auto-trak *	Insp. T. Min/Max (optional) Insp. Time	Insp. Time
Air Liquide (Anthony, France)	EO 150	Exp. Trig.	90% to 10% or Auto *	I Time Min and I Time Max Backup I. Time	Backup I. Time or according to Exp. Trig. if set on Auto

For the ranges of the TgE, the percentage values represent the percentages of the peak inspiratory flow. ∫ In these devices, the TgE is set as a numerical value (1–9), with 1 being the shortest TgE, corresponding to 90% of peak inspiratory flow. * The true automatic setting of the TgE. Abbreviations: TgE, expiratory trigger sensitivity; Ti, inspiratory time; min, minimal; max, maximal.

**Table 2:** Cycling asynchronies in spontaneous/timed mode.

	Early Cycling	Delayed Cycling
Causes	Too short TgE (high percentage of peak inspiratory flow) Short Ti min (restrictive diseases) Short Ti max Short backup Ti or Ti timed (controlled breaths)	Too long TgE (low percentage of peak inspiratory flow) Long Ti min Unintentional leaks (with long Ti max) High backup Ti or Ti timed (controlled breaths)

Abbreviations: TgE, expiratory trigger sensitivity; Ti, inspiratory time; min, minimal; max, maximal.

## Data Availability

The datasets generated and/or analyzed during the current study are available from the authors on reasonable request.
